# A comparative approach to testing hypotheses for the evolution of sex‐biased dispersal in bean beetles

**DOI:** 10.1002/ece3.1753

**Published:** 2015-10-08

**Authors:** Michelle H. Downey, Rebecca Searle, Sunil Bellur, Adam Geiger, Brian S. Maitner, Johanna R. Ohm, Midori Tuda, Tom E. X. Miller

**Affiliations:** ^1^ Department of BioSciencesProgram in Ecology and Evolutionary BiologyRice UniversityHoustonTexas77005; ^2^ Department of Ecology and Evolutionary BiologyUniversity of ArizonaTucsonArizona85721; ^3^ Department of BiologyCenter for Infectious Disease DynamicsPennsylvania State UniversityUniversity ParkPennsylvania16803; ^4^ Laboratory of Insect Natural EnemiesDivision of Agricultural Bioresource SciencesDepartment of Bioresource SciencesFaculty of AgricultureKyushu UniversityFukuoka812‐8581Japan; ^5^ Institute of Biological ControlFaculty of AgricultureKyushu UniversityFukuoka812‐8581Japan

**Keywords:** Bean beetle, inbreeding, mating system, polygyny, sex‐biased dispersal

## Abstract

Understanding the selective forces that shape dispersal strategies is a fundamental goal of evolutionary ecology and is increasingly important in changing, human‐altered environments. Sex‐biased dispersal (SBD) is common in dioecious taxa, and understanding variation in the direction and magnitude of SBD across taxa has been a persistent challenge. We took a comparative, laboratory‐based approach using 16 groups (species or strains) of bean beetles (genera *Acanthoscelides*,* Callosobruchus*, and *Zabrotes*, including 10 strains of one species) to test two predictions that emerge from dominant hypotheses for the evolution of SBD: (1) groups that suffer greater costs of inbreeding should exhibit greater SBD in favor of either sex (inbreeding avoidance hypothesis) and (2) groups with stronger local mate competition should exhibit greater male bias in dispersal (kin competition avoidance hypothesis). We used laboratory experiments to quantify SBD in crawling dispersal, the fitness effects of inbreeding, and the degree of polygyny (number of female mates per male), a proxy for local mate competition. While we found that both polygyny and male‐biased dispersal were common across bean beetle groups, consistent with the kin competition avoidance hypothesis, quantitative relationships between trait values did not support the predictions. Across groups, there was no significant association between SBD and effects of inbreeding nor SBD and degree of polygyny, using either raw values or phylogenetically independent contrasts. We discuss possible limitations of our experimental approach for detecting the predicted relationships, as well as reasons why single‐factor hypotheses may be too simplistic to explain the evolution of SBD.

## Introduction

The study of dispersal is important for many reasons. Among them are the predictions of expansion by invasive species (Hastings et al. 2005), understanding historical drivers of species' distributions (Clark 1998) and responses of species ranges to ongoing climate change (Dullinger et al. 2004). Patterns of movement differ among species and even among individuals of the same species, including age‐, stage‐, and sex‐specific movement (Neubert and Caswell 2000; Miller et al. 2011). A major goal of evolutionary ecology is to understand the selective forces that have shaped variation in dispersal behavior among and within species (Ronce 2007).

In dioecious taxa (those with separate sexes), dispersal is commonly sex‐biased (Greenwood 1980; Pusey 1987; Miller et al. 2011; Dobson 2013). The direction and magnitude of sex bias in dispersal varies across taxa and may be associated with taxonomic groups and / or mating systems. For example, dispersal tends to be female‐biased in monogamous taxa such as birds and male‐biased in promiscuous taxa such as mammals (Greenwood 1980; Pusey 1987; Handley and Perrin 2007; Dobson 2013). Sex bias in dispersal can have important implications for the spread of invasive organisms (Miller et al. 2011; Miller and Inouye 2013) and for the genetic structure of metapopulations (Kerth et al. 2002; Fraser et al. 2004). Understanding the selective forces that favor sex‐biased dispersal has been a persistent challenge (Handley and Perrin 2007; Dobson 2013).

There are two leading hypotheses to explain the evolution of sex‐biased dispersal. First, costs of inbreeding can select for dispersal as a strategy to avoid mating with kin and its detrimental fitness effects due to the expression of deleterious alleles (Gandon 1999; Perrin and Mazalov 1999; Roze and Rousset 2005). This mechanism of inbreeding avoidance requires only that one sex disperse while the other may remain philopatric; in the absence of sex‐specific costs of inbreeding, dispersal bias in favor of either females or males is equally likely (Perrin and Mazalov 1999). As the negative fitness effects of inbreeding become stronger, so should selection for sex‐biased dispersal as an inbreeding avoidance strategy. Second, competition among relatives, or kin competition, can select for directional sex bias in dispersal. Male‐biased dispersal is expected to enhance inclusive fitness when related males compete for access to females (“local mate competition”), while female‐biased dispersal should be favored when related females compete for access to resources necessary for rearing offspring such as food or nesting sites (“local resource competition”) (Perrin and Mazalov 2000). The kin competition hypothesis could explain associations between sex‐biased dispersal and mating system. For example, polygynous mating systems (where a single male can fertilize multiple females) are typically associated with strong male competition for females. The occurrence of male‐biased dispersal in taxa with polygynous mating systems is therefore consistent with the kin competition hypothesis, because male dispersal would enhance inclusive fitness, all else equal (Greenwood 1980; Handley and Perrin 2007; Dobson 2013). Thus, the kin competition hypothesis predicts that sex‐biased dispersal reduces competition between relatives, although it need not reduce competition overall. The inbreeding avoidance and kin competition hypotheses are not mutually exclusive. While inbreeding avoidance, alone, is not expected to select for a particular direction of sex bias in dispersal, it may amplify selection in the direction set by kin competition (Perrin and Mazalov 1999, 2000).

Current understanding of the selective forces underlying sex differences in dispersal come predominantly from verbal and mathematical theory. Empirical understanding of the drivers of sex‐biased dispersal lags behind theory. Many empirical studies focus on individual taxa, examining qualitative trait associations with sex‐biased dispersal. For example, studies have tested whether taxa with high inbreeding potential exhibit sex‐biased dispersal (Kerth et al. 2002), or whether taxa that are polygynous exhibit male‐biased dispersal, as predicted by the kin competition hypothesis (e.g., Hutchings and Gerber 2002; Cutrera et al. 2005; Nagy et al. 2007; Cano et al. 2008; Innocent et al. 2010). To our knowledge, quantitative relationships between sex‐biased dispersal and inbreeding or kin competition have not been previously explored in a comparative context. Furthermore, most existing comparative studies do not employ appropriate phylogenetic controls, which is necessary to quantify variation in sex‐biased dispersal across taxa (Perrin and Mazalov 1999; Handley and Perrin 2007). For example, the prevalence of female‐biased dispersal in birds could be more a reflection of shared phylogenetic constraint than of shared selective pressures related to mating system.

In this study, we used a comparative phylogenetic approach to test the following two predictions about sex‐biased dispersal (not absolute dispersal distance) that emerge from theory. First, we predicted that taxa that suffer greater costs of inbreeding should exhibit greater sex bias in dispersal. Because inbreeding alone does not select for a direction of dispersal bias in the absence of sex‐specific costs (Perrin and Mazalov 1999), we examined the relationship between inbreeding depression and the absolute value of dispersal bias (bias in either direction). Second, we predicted that taxa with stronger kin competition should exhibit greater bias in dispersal. We specifically focus on competition between related males for mating opportunities (local mate competition), based on the natural history of our study system. Our work focused on a clade of bean beetles (family: Chrysomelidae, subfamily: Bruchinae) for which we have a well‐resolved phylogeny, including seven species from three genera and ten genetically distinct populations of one species. This group, which includes pests of stored beans, has emerged as a valuable experimental system for the study of dispersal (Strevens and Bonsall 2011; Miller and Inouye 2013). Because this group is known to be highly polygynous (Arnqvist et al. 2005; Miller and Inouye 2011, 2013), we expected kin competition to be stronger among males than females; therefore, we focus on local mate competition and not local resource competition. In addition, prior work with bean beetles demonstrated male‐biased dispersal (Miller and Inouye 2013), further suggesting a potential role for local mate competition. We therefore had an a priori expectation of a qualitative association between polygyny and male‐biased dispersal across beetle groups. Here, we ask for the first time whether quantitative variation in the degree of male bias can be explained by inbreeding depression or potential for local mate competition.

We used laboratory experiments to quantify sex bias in dispersal, the primary response variable, and two potential predictor variables: costs of inbreeding, and degree of polygyny (number of female mates per male). We interpret the degree of polygyny (number of female mates per male) as a proxy for local mate competition, because competition between related males for mating opportunities should increase with potential for single males to fertilize multiple females. We then used Bayesian statistical methods and phylogenetically independent contrasts (PICs) to test the strength of the predicted trait relationships.

## Methods

### Study organisms

Bean beetles complete their life cycle entirely on dry beans. For most species, eggs are deposited on the surface of beans (one of our focal species, *Acanthoscelides obtectus*, oviposits near but not on beans). Larvae develop inside the bean, and adults emerge, mate and die without requiring any additional food or water. The complete life cycle takes ca. 32 days under our laboratory conditions (27.5°C and a 16:8 h photoperiod). Adults are sexually dimorphic and distinguishable by shape, size, and coloration. All beetles used in our experiments were raised on black‐eyed peas (*Vigna unguiculata*).

The beetle groups used in this experiment are listed in Table 1. They include the genera *Acanthoscelides*,* Callosobruchus*, and *Zabrotes*, five species of *Callosobruchus*, and 10 populations of *C. maculatus* for a total of 16 “groups” (species or populations of *C. maculatus*). The *C. maculatus* populations were collected from localities distributed worldwide (this species is a cosmopolitan pest of legumes) and are known from previous work to exhibit significant genetic and phenotypic differentiation (Dowling et al. 2007; Rankin and Arnqvist 2008; Arnqvist and Tuda 2010; Tuda et al. 2014). Thus, the groups used in this study provide broad phenotypic and phylogenetic coverage, including interpopulation, interspecies, and intergenus variation. At the time of our experiments, each beetle group had been maintained under laboratory conditions for at least 50 generations and likely many more. These beetle lines were thus well adapted to laboratory conditions but nonetheless maintained significant trait variation, even among populations of *C. maculatus* (e.g., Arnqvist and Tuda 2010). Beetle lines were shared with us by the laboratories of G. Arnqvist, Y. Toquenaga, and C. Fox.

**Table 1:** Bean beetle groups (Coleoptera: Chrysomelidae [Bruchinae]) used for comparative analyses of sex‐biased dispersal evolution

Species	Population	Abbreviation
*Acanthoscelides obtectus*	−	ACOB
*Callosobruchus chinensis*	−	CACH
*Callosobruchus maculatus*	Benin	BENI
*Callosobruchus maculatus*	Brazil	BRAZ
*Callosobruchus maculatus*	California	CALI
*Callosobruchus maculatus*	IITA	IITA
*Callosobruchus maculatus*	India	INDI
*Callosobruchus maculatus*	Mali	MALI
*Callosobruchus maculatus*	Nigeria	NIGE
*Callosobruchus maculatus*	Uganda	UGAN
*Callosobruchus maculatus*	Upper Volta Burkina Faso	UVBF
*Callosobruchus maculatus*	Yemen	YEME
*Callosobruchus phaseoli*	–	CAPH
*Callosobruchus rhodesianus*	–	CARH
*Callosobruchus subinnotatus*	–	CASU
*Zabrotes subfasciatus*	–	ZASU

### Phylogenetic relationships

**Figure 1:**
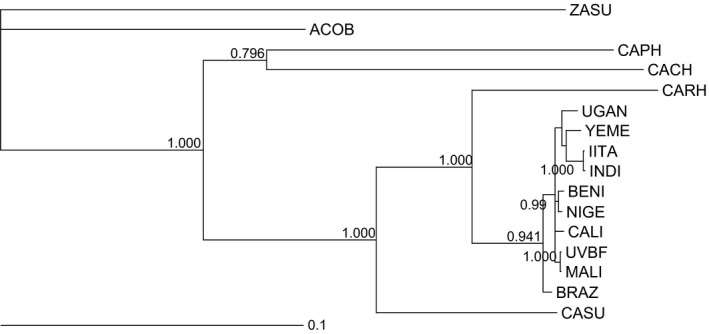
Phylogenetic relationships of 16 focal groups (Table 1) based on two mitochondrial markers (cytochrome *c* oxidase subunits I and II) and one nuclear marker (28S rRNA). Branch length legend is in the units of expected substitutions per nucleotide site. Figure shows majority rule consensus Bayesian tree (unrooted). Only posterior probabilities >0.7 are shown.

Phylogenetic information for all 16 focal groups came from previous work by M. Tuda and colleagues (Tuda et al. 2006, 2014). The phylogeny was constructed using sequence data from mitochondrial (cytochrome *c* oxidase subunits I and II) and nuclear (28S rRNA) markers under a general time reversible model of evolution with gamma‐distributed rate variation (GTR+G model) in a Bayesian framework (details in Tuda et al. 2006, 2014). The resulting phylogeny is shown in Figure 1.

### Sex‐biased dispersal experiment

Our dispersal experiments focused on crawling movement. Some of the beetle groups in our study are known to exhibit flight dimorphisms involving flight‐capable and flight‐incapable morphs; this is best documented in *C. maculatus* (Utida 1972; Messina and Renwick 1985). Therefore, it may be important to consider both dispersal modes. Indeed, our original study design included comparisons of crawling and flight dispersal across beetle groups. However, our preliminary behavioral observations (ten individuals per beetle group observed for 5 min) indicated that flight propensity was very low: only *C. rhodesianus* and *C. subinnotatus* exhibited any propensity for flight. Thus, flight observations did not yield much information that could be analyzed in a comparative context. For this reason, we focus on crawling. However, flight is a complex trait that is at least partly heritable (Sano‐Fujii 1986) and also inducible by environmental conditions such as crowding (Utida 1972) and water availability (Sano‐Fujii 1984). It is possible that our laboratory rearing conditions suppressed the production of flight‐capable beetles, masking variation across groups.

We tested crawling dispersal ability using artificial landscapes of Petri dishes connected by 1 cm of plastic tubing. The one‐dimensional dispersal arrays consisted of 41 connected patches (dishes), each of which contained 5 g of black‐eyed peas. For each dispersal trial, ten newly emerged (<48 h old) beetles (five females, five males) were released into the center patch of the array and allowed to disperse in either direction. Trials ran for 24 h, after which the patch locations of females and males were recorded (locations of any individuals that died during the trial were excluded from analyses). Each group had a minimum of 6 trials, for a minimum of 30 observed dispersal distances for each sex (a maximum of 55 female observations, 37 male observations, and an average of 39 female observations and 33 male observations). We distributed the total number of dispersing beetles across multiple trials to minimize potential for local density to influence dispersal distance.

To characterize the occurrence and magnitude of sex bias in dispersal, we fit Poisson dispersal distributions (or “kernels”) to sex‐specific dispersal data (absolute value of the number of patches moved). The Poisson is an appropriate kernel for discrete landscapes as it relies on a single parameter for the mean and variance of dispersal distance, providing a convenient metric to compare female and male kernels. We characterize the direction and degree of sex bias using the log ratio of mean female dispersal distance to mean male dispersal distance $\left({\text{log}}_{e}\left(\frac{\mu_{F}}{\mu_{M}}\right)\right)$. This quantity takes positive values for female bias, negative values for male bias, and a value of zero for identical dispersal between the sexes. We fit dispersal kernels to data using Bayesian statistical software (JAGS 3.4.0 and the R interface R2jags: Su and Yajima 2014) that samples from the posterior probability distribution of model parameters (in our case, Poisson means) with Markov chain Monte Carlo (MCMC) methods. The advantage of a Bayesian approach is that it allows us to incorporate the uncertainty in the sex‐specific dispersal means into the uncertainty in the log ratio, a quantity derived from them. We evaluated the statistical significance of sex bias based on whether the Bayesian credible interval for this quantity excluded zero. The Bayesian approach also allows us to appropriately account for uncertainty in the analysis of trait correlations (below).

### Inbreeding experiment

We created experimental crosses within and between maternal families to quantify the effects of inbreeding across groups. We employed a block design following Fox et al. (2011), where each block was composed of two maternal families, initiated with randomly selected females from their respective stock populations. Within each block, there were four experimental crosses, two outbred crosses (female from family A × male from family B or vice versa) and two inbred crosses (female from family A × male from family A and female from family B × male from family B). We replicated this block design three times for all groups except *C. phaseoli*, which was not included in the inbreeding experiment (it was unavailable at the time of the experiment). The offspring of these crosses were counted and weighed. We focus here on offspring number as the response variable, which assumes that any inbreeding depression is manifest in survival from the egg stage through adult eclosion and emergence. Results were qualitatively identical when we instead analyzed offspring mass.

For each block, we estimated inbreeding depression as offspring production in the two inbred crosses minus offspring production in the two outbred crosses and divided the difference by the outbred value (Fox et al. 2011). This quantity is thus the proportional change in offspring production due to inbreeding. We estimated inbreeding depression for each group using JAGS, with offspring number modeled as a Poisson variable for inbred and outbred crosses. This analysis included two random effects: the two‐family blocks within each taxon and the six unique maternal families within each block; the latter random effect accounts for the non‐independence between, for example, the crosses female A × male A and female A × male B.

### Mating system experiment

Finally, we estimated variation in mating system (degree of polygyny) across groups. We quantified mating system indirectly by examining how experimental variation in sex ratio affected one generation of population growth. The population growth of more polygynous groups should be less affected by female‐biased sex ratios, because fewer males would be needed to fertilize all females. We used a response surface experimental design (Miller and Inouye 2011) that crossed initial female density (1 to 9) with initial male density (1 to 9) for a total of nine female × male combinations, each replicated two times. Each replicate population began with virgin (<48 h after emergence) beetles placed in Petri dishes with five grams of black‐eyed peas. The response variable was the total number of recruits in the next generation.

We fit to these data a two‐sex demographic model in which recruitment in the next generation (*N*_*t+1*_) was proportional to the harmonic mean of female (F_*t*_) and male (M_*t*_) densities in the starting generation:

(1) $$N_{t+1}=\lambda \frac{2F_{t}M_{t}}{\frac{F_{t}}{h}+M_{t}}$$

This the harmonic mean function for two‐sex recruitment is well established in the demography literature (Lindström and Kokko 1998; Caswell 2001; Miller et al. 2011), and a previous study showed that it provided a good fit to beetle recruitment data (Miller and Inouye 2011). Parameter *λ* represents the reproductive rate. Differences in baseline fertility across beetle groups are captured by *λ*. The main parameter of interest is the harem size, *h*, which determines how population growth responds to variation in sex ratio. This parameter thus provides an indirect inference about the mating system. Under strict monogamy (*h *=* *1), per capita population growth is maximized at a 1:1 sex ratio, where every male has a single female mate. Under this condition, competition among males for access to females, and hence local mate competition, should be weak or absent as long as the birth sex ratio is also 1:1, as it is in bean beetles (Miller and Inouye 2013). As *h* increases, population growth is maximized under increasingly female‐biased sex ratios because fewer males are required to saturate female mating opportunities (Miller and Inouye 2011), in which case local mate competition should increase. Thus, the harem size parameter approximates the degree of polygyny and hence potential for competition among related males for female mates. We fit Eq. (1) to data and estimated the harem size for each taxon using JAGS.

### Testing trait correlations

We predicted positive correlations across groups between sex‐biased dispersal and the negative effects of inbreeding (groups that exhibit greater negative effects of inbreeding also exhibit greater sex bias in either direction [toward either females or males]), and between sex‐biased dispersal and the degree of polygyny (more polygynous groups exhibit greater male bias). To test these correlations, we estimated all traits within a single Bayesian framework, then estimated the posterior probability distribution of Pearson correlation coefficients (*r*) corresponding to the joint posterior probability distribution of the trait values. We tested the statistical significance of the correlations by asking whether the 95% credible intervals included zero. For the inbreeding/dispersal correlation, we used the absolute value of the log ratio of mean female‐to‐mean male dispersal distances $\left(|{\text{log}}_{e}\left(\frac{\mu_{F}}{\mu_{M}}\right)|\right)$, thus capturing bias in either direction. Each draw from the joint posterior, representing one MCMC iteration, was associated with one pair of correlation coefficients (for the two trait pairs). By collecting all draws from the joint posterior, we are better able to capture uncertainty in the trait correlations. The alternative approach – calculating a single correlation coefficient for one set of trait values (means) – would assume that traits are invariant across individuals and measured without error. Thus, the Bayesian approach allowed us to propagate uncertainty in the underlying trait values to uncertainty in the correlation coefficient.

### Phylogenetically independent contrasts

In addition to the raw trait correlations, we also used phylogenetically independent contrasts (PICs) to control for any biases in the correlation results due to shared evolutionary history (Felsenstein 1985). The R Package ape (v3.1‐4, http://ape-package.ird.fr/) was used to calculate independent contrasts for the variables. To confirm correct standardization of contrasts (evolution consistent with a Brownian motion model), we used diagnostic scripts included in the R package picante (http://ib.berkeley.edu/courses/ib200b/scripts/ diagnostics_v3.R) which suggested an untransformed tree was appropriate. For the inbreeding analysis, we excluded *C. phaseoli*.

As in the analysis of raw trait values, we used a Bayesian approach to estimate a posterior probability distribution for the correlation of PIC values across groups. For each draw from the posterior distribution of trait values, we calculated PICs for sex‐biased dispersal, inbreeding depression, and polygyny, and their pairwise correlation coefficients (dispersal vs. inbreeding and dispersal vs. polygyny). The resulting distribution of correlation coefficients therefore reflects uncertainty in the strength of the relationship given uncertainty in trait estimation and controlling for phylogeny (but not accounting for uncertainty in the phylogeny itself).

Finally, to test whether combinations of polygyny and inbreeding depression could better explain observed sex‐biased dispersal than either factor alone, we fit linear models to group mean trait values. We fit candidate models that included inbreeding depression (proportional effect of inbreeding on offspring production) and degree of polygyny (natural log of estimated harem size) as predictor variables (both traits alone, both traits together as additive or interactive, and a null model with no predictor variables, for five models in total). The response variable was the log ratio of female‐to‐male dispersal ${\text{log}}_{e}\left(\frac{\mu_{F}}{\mu_{M}}\right)$. Note that because this analysis used Bayesian posterior trait means, there was no replication at the level of beetle groups (quantities for dispersal bias and degree of polygyny were inferred from lower‐level observations; we therefore do not have replicate estimates by beetle group). Therefore, we could not include random effects associated with phylogenetic relationships, because these effects are indistinguishable from residual variance. The analysis therefore does not account for phylogeny and should be interpreted with appropriate caution. We used likelihood ratio tests to determine whether the two explanatory traits, alone or in combination, provided a better fit than the null model.

## Results

### Trait variation

**Figure 2:**
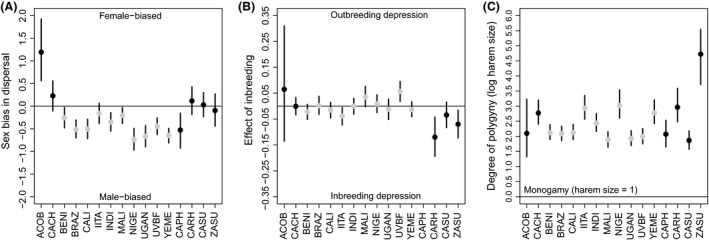
Trait variation across groups. (A) Sex bias in dispersal (log ratio of the mean female‐to‐mean male dispersal distance). Positive and negative values indicate female‐ and male‐biased dispersal, respectively. Horizontal line shows no bias. (B) Effect of inbreeding (proportional effect of inbreeding on offspring recruitment relative to an outbred cross). Positive and negative values indicate positive and negative effects of inbreeding, respectively. Horizontal line shows no effect. (C) Degree of polygyny (log number of female mates per male (harem size)). Horizontal line shows monogamy (*h* = 1). Taxon codes on the x‐axis correspond to Table 1. Points show posterior means, and bars show 95% credible intervals (may be obscured by points in C). Gray points represent ten populations of *C. maculatus*. All other points represent distinct species.

We found significant variation in all three traits across groups. Beetles dispersed a minimum of 0 patches and a maximum of 20 patches, with a mean of 4.1 patches. Dispersal ranged from significantly female‐biased in one group (*A. obtectus*) to significantly male‐biased in ten other groups, including most populations of *C. maculatus* and the species *C. phaseoli*. There was no significant dispersal bias (credible intervals for the log ratio of female‐to‐male dispersal included zero) in five groups (Fig. 2A). Even among groups that exhibited significant sex bias, there were quantitative differences in magnitude. For example, the Mali and Yemen populations of *C. maculatus* both exhibited significant male bias, but the degree of bias of Yemen beetles was more than twice that of Mali beetles (Fig. 2A).

We also found variation across groups in the effects of inbreeding on offspring recruitment (Fig. 2B). Offspring production ranged from a minimum of three recruits (in *A. obtectus*) to a maximum of 125 (in *C. maculatus* (Benin)), with a mean of 55. However, only three of the groups we tested (*C. maculatus* (IITA), *C. rhodesianus* and *Z. subfasciatus*) exhibited significant inbreeding depression (the credible interval for the proportional effect of inbreeding was below zero). One group (*C. maculatus* (UVBF)) showed a positive response to inbreeding (i.e., outbreeding depression).

Finally, we found variation in mating systems across groups (Fig. 2C). All of the bean beetle groups we tested were significantly polygynous (i.e., all 95% credible intervals for harem size exceeded 1, which corresponds to perfect monogamy). There was variation within and across species in estimated harem size, although most were below 30 female mates per male. One group (*Z. subfasciatus*) was a strong outlier, with an estimated harem size of 125 females and a very wide credible interval (it appears less extreme on the log scale of Figure 2C). We re‐inspected the raw data and concluded that the high estimate for harem size is likely correct. This species showed little reduction in female fertility in highly female‐biased populations, suggesting that few males can fertilize many females and hence the inference of very strong polygyny.

### Trait correlations

**Figure 3:**
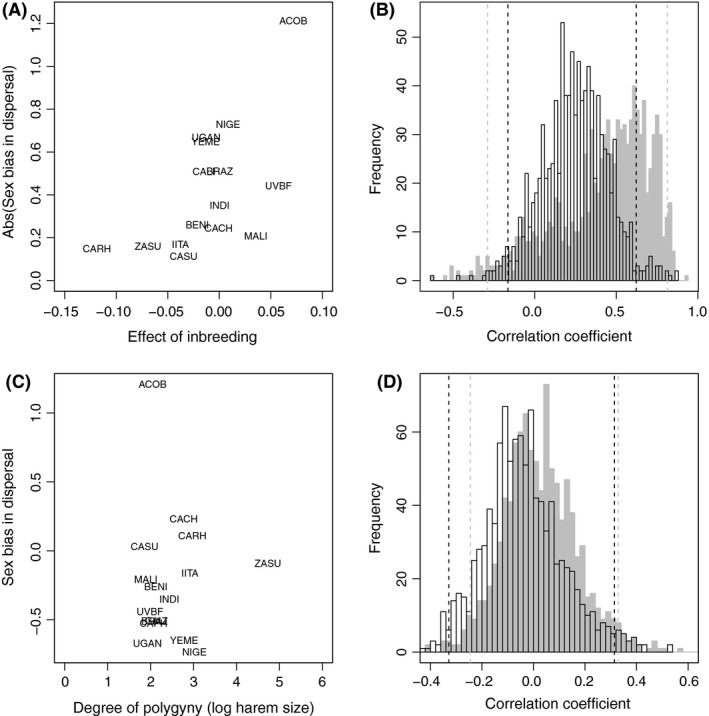
Traits correlations across groups. (A, C) Joint posterior means for (A) sex bias in dispersal (absolute value of the log ratio of female‐to‐male dispersal distance, indicating bias in either direction) and effect of inbreeding (proportional effect of inbreeding on offspring recruitment), and (C) sex bias in dispersal (signed value of the log ratio of female‐to‐male dispersal distance) and degree of polygyny (log number of female mates per male, or harem size). Locations of 4‐letter taxon symbols (Table 1) show bivariate means. (B, D) Posterior probabilities for correlation between absolute dispersal bias and inbreeding effect (B) and signed dispersal bias and polygyny (D). Gray bars show the correlation of raw trait values, and unfilled bars show the correlation of the phylogenetically independent contrasts. Vertical dashed lines represent bounds of the 95% credible intervals for the correlations of raw trait values (gray) and phylogenetically independent contrasts (black).

There was no evidence for statistical association between the magnitude of inbreeding depression and the magnitude of sex bias in dispersal (toward either females or males) based on raw (phylogenetically uncorrected) trait values (Fig. 3A and B). In fact, the trait correlation tended toward the opposite direction than we predicted: The groups that exhibited the strongest sex bias in dispersal were those that suffered least (or even benefitted) from inbreeding (mean correlation: 0.43; Fig. 3A). However, the posterior probability distribution for the correlation coefficient included zero in its 95% credible interval (Fig. 3B). Accounting for the shared phylogenetic history of the beetle groups did not modify this result. The mean correlation coefficient for the PICs of inbreeding depression and sex bias in dispersal was 0.22 and the credible interval included zero, as it did for the raw trait value correlation (Fig. 3B).

Likewise, we found no evidence for an association between polygyny and male‐biased dispersal based on raw trait values (Fig. 3C). The mean correlation coefficient was 0.03 and the credible interval for this correlation included zero (Fig. 3D). Again, accounting for phylogeny did not modify this result. The mean correlation coefficient for the PICs of inbreeding depression and sex bias in dispersal was −0.06 and the credible interval included zero (Fig. 3D).

Finally, consistent with the pairwise, phylogenetically controlled correlation results, we found no support for linear models that included combined effects of inbreeding and polygyny on sex‐biased dispersal. Among five candidate models that included effects of inbreeding and degree of polygyny as predictor variables, alone or in combination, the null model (no influence of either trait) received the most AIC support (AIC weight = 0.46) and no other model provided a significant improvement based on likelihood ratio tests (all *P *>* *0.25).

## Discussion

Comparative approaches have played an important role in understanding the selective forces that shape patterns of dispersal across taxonomic groups, including Greenwood's now‐classic observation of associations between mating system and sex‐biased dispersal (Greenwood 1980; Dobson 2013). We used a comparative approach with 16 groups of bean beetles to evaluate support for hypothesized associations of sex‐biased dispersal with inbreeding depression and local mate competition, two factors expected to play a role in dispersal evolution. Subsets of our data were consistent with certain predictions; for example, qualitatively, male‐biased dispersal and polygyny were pervasive among the bean beetle groups (Fig. 2), consistent with the prediction that male‐biased dispersal can enhance inclusive fitness when related males compete for access to females. However, in total, our comparative experiments failed to support quantitative relationships between sex‐biased dispersal and either inbreeding depression or kin competition.

One interpretation of our results is that the inbreeding and kin competition hypotheses, alone, are insufficient to fully explain variation in sex‐biased dispersal. Indeed, several recent studies report results that are similarly inconsistent with predictions. For example, in contrast to predictions from the kin competition hypothesis, Hammond et al. (2006) found evidence for female‐biased dispersal in a highly polygynous primate with local mate competition among males; they suggest that kin competition, inbreeding avoidance, and social structure could interact in complex ways to shape the female‐biased dispersal strategy. Similarly, Duarte et al. (2003) found female‐biased dispersal in shrews despite evidence for polygyny and no negative fitness effects of inbreeding. These authors suggest that benefits of familiarity with natal territory could promote male philopatry or that local extinction/colonization dynamics could favor female dispersal as means to establish social groups in open habitat.

One limitation of our approach is that we focus on local mate competition between related males as the sole source of kin competition. Resource competition between related females is an unmeasured factor that would act in the opposite direction, favoring female‐biased dispersal. Indeed, we found evidence for female‐biased dispersal in one species (*A. obtectus*). Better understanding of the natural history of this species could help determine whether there is reason to expect local resource competition as an important evolutionary force. In general, the selective environments of bean beetles' recent evolutionary histories (infestation of stored grains or field crops) and their status as pests suggest that resources have not been strongly limiting. For this reason, we continue to expect greater potential for kin competition between males (for mates) than females (for oviposition resources), although mate competition is clearly not a sufficient explanation for the patterns of variation that we observed.

Recent theory identifies additional demographic factors, not necessarily tied to inbreeding or kin competition, that can contribute to dispersal differences between the sexes. For animals dispersing to locate mates, costs of dispersal would favor only one sex dispersing, such that the other avoids unnecessary dispersal costs (Meier et al. 2011; Shaw and Kokko 2014). Factors such as landscape heterogeneity, sex‐specific mortality, and the timing of mating during the dispersal process can also affects the selective costs and benefits of sex‐specific movement (Shaw and Kokko 2014). Thus, these and other factors may have played an important role in the evolutionary history of our focal groups, possibly overwhelming the roles of single factors like inbreeding or kin competition. As new models of dispersal evolution begin to tackle realistic aspects of the dispersal process and its ecological contexts (Shaw and Kokko 2014), evolutionary ecologists may need to revisit simple, long‐standing hypotheses for the selective advantages of sex‐biased dispersal. In addition, Greenwood's classic paper (Greenwood 1980) suggests that advantages of sex‐biased dispersal must be considered against the advantages of philopatry, which will often be specific to the biology of the focal species. For example, the ability of males to acquire resources in their natal habitat or advantages of habitat familiarity could counteract selection for male‐biased dispersal (Greenwood 1980; Duarte et al. 2003). Benefits of philopatry in bean beetles are not well known but merit further study.

Our laboratory‐based approach with “captive” populations of bean beetles provided several advantages regarding taxonomic breadth of the study and power to estimate the values of traits that are very difficult to rigorously quantify in natural populations. However, this approach also involved drawbacks that may have clouded our ability to detect the hypothesized relationships. Our study organisms have been maintained in a laboratory environment for many generations. The selective regime of the laboratory may have altered dispersal behavior relative to wild beetles. For example, the costs and benefits of dispersal behavior in a laboratory context are both likely to be minimal, in which case selection on dispersal would be very weak and dispersal traits may change simply due to drift. Such an influence of the recent selective environment may override any historical contributions of inbreeding depression or kin competition to dispersal evolution. Previous studies have used some of the same beetle populations to test the adaptive significance of trait variation across groups (Rankin and Arnqvist 2008; Arnqvist and Tuda 2010), so the methodological premise of our study has precedent. Indeed, the fact that we observed male‐biased dispersal across many beetle groups (Fig. 2A) suggests that, even in lab culture, they have retained traits reflective of their deeper evolutionary history. However, we do not know how well the dispersal behavior we observed in our assay (crawling through tubes) corresponds to dispersal in the wild, which may involve flight for some of the groups. Our preliminary observations indicated very low flight propensity across our focal groups. However, it is possible that flight decisions are context dependent or that flight morphology and behavior are environmentally inducible (Messina and Renwick 1985). A careful examination of the role of flight dispersal in these beetles, perhaps under various rearing conditions, could be a valuable next step.

It is also likely that maintenance in laboratory culture has altered the consequences of inbreeding depression relative to conditions in natural populations. The genetic bottleneck associated with establishment of the laboratory stocks, followed by many generations of inbreeding, likely purged much of the genetic load in our focal populations. Indeed, we found very little evidence for negative effects of inbreeding (Fig. 2B), consistent with this hypothesis. Follow‐up experiments that conduct hybridization crosses between independent strains of the same species could help determine whether previous purging of deleterious alleles could explain our results. In contrast to our results, previous studies with laboratory‐maintained lines of bean beetles have detected significant inbreeding depression using similar experimental designs (e.g., Fox et al. 2008; Fox and Reed 2011). Fox and Reed (2011) showed that inbreeding depression in laboratory‐maintained lines of *C. maculatus* increased with environmental stress and that, across diverse groups, inbreeding depression may be difficult to detect in benign environments. Thus, a more challenging plant host or abiotic context may yield different quantitative estimates for the effects of inbreeding in these groups.

In summary, our work provides a thorough and phylogenetically explicit investigation of sex‐biased dispersal across related species and populations. The results add to our growing understanding of variability in the direction and magnitude of sex‐biased dispersal. Yet, the evolutionary forces that may explain this variability across bean beetles remain elusive and merit continuing study.

## Conflict of Interest

None declared.
